# Recipients of 2022 CSSA Editor's Citation for Excellence Named

**DOI:** 10.1002/tpg2.20344

**Published:** 2023-04-21

The editorial board of *The Plant Genome* is pleased to announce the recipients of the 2022 Editor's Citation for Excellence. These awards recognize the outstanding professional commitment and dedication of volunteer reviewers and/or editors who, through their excellent insights and comments, have helped maintain the high standard and quality of papers published in the journal. Recipients were nominated based on their thorough, competent, and timely reviews or editing of manuscripts. Each will receive a certificate of appreciation, an ASA‐CSSA‐SSSA gift certificate, and recognition in CSA News.

## Associate Editors


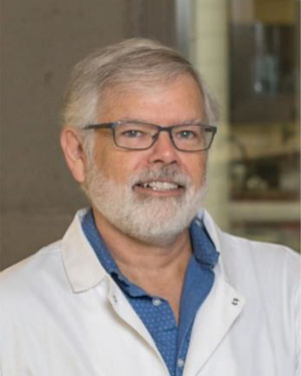
Dr. François Belzile was trained as a plant molecular geneticist and initially worked on DNA repair and recombination genes in the model plant *Arabidopsis thaliana*. Over the last 20 years, Dr. Belzile's lab at the Université Laval, Canada, has been interested in using genomics to develop novel approaches in plant breeding, mainly in soybean and barley. Dr. Belzile aims to act as a bridge and a facilitator between the lab‐based genomic sciences and their numerous applications in the development of new and improved crop varieties. His lab works closely with industry partners to jointly develop and implement genomics‐informed breeding programs.


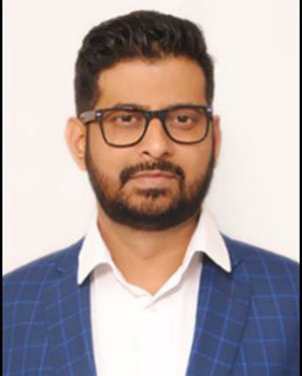
Dr. Rupesh Deshmukh is currently working as Associate Professor at the Central University of Haryana, India. He obtained his PhD degree in Agriculture Biotechnology from SRTMU, Nanded, India. His group is working on improving rice and tomato crops through integrated genomic approaches. Crop improvement for nutritive food and sustainable agriculture are two major areas of his research. His name is featured in a list of the World's Top 2% of highly cited researchers published by Stanford University. He received several highly competitive awards including Ramalingaswami Re‐entry Fellowship and NASI‐Scopus Young Scientist Award. He is recognized as a Fellow of the ISGPB and ARRW scientific societies.

## Reviewers


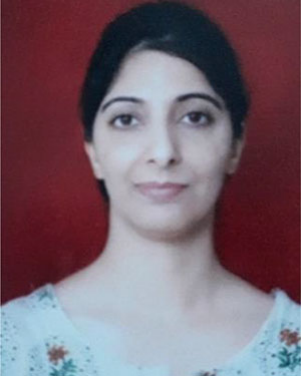
Dr. Humira Sonah earned her master's degree in Agriculture Biotechnology from Indira Gandhi Agriculture University, Raipur, India. She did her Doctorate from Banasthali University while working at the National Institute on Plant Biotechnology. Soon after her Ph.D., she joined Laval University, Canada, as a Postdoctoral Researcher where she was involved to explore the genotyping‐by‐sequencing method useful to accelerate crop improvement followed by Genome‐wide association studies. She did another postdoctoral fellowship at Missouri University, USA. She rejoined Laval University and worked as a Visiting Professor. Considering her remarkable achievements in Plant Biology, the Department of Biotechnology (DBT), Government of India awarded her Ramalingaswami Fellowship which is one of the most prestigious awards aiming to attract high‐quality Indian nationals working abroad. Presently, Dr. Sonah is Ramalingaswami Fellow at National Agri‐Food Biotechnology Institute and her group is working to develop food‐grade soybean. She was featured in 75 under 50, Scientist shaping today's India‐2022 published by Vigyan Prasar, Government of India on National Science Day, and also featured in the list of world's top 2% researchers published by Stanford University (2022).

Not pictured: Dr. Philipp Bayer, The University of Western Australia

